# 3-Month Outcome of Ischemic Stroke Patients Underwent Thrombolytic Therapy; a Cohort Study

**Published:** 2020-01-25

**Authors:** Payam Sari Aslani, Shahab Rezaeian, Elham Safari

**Affiliations:** 1 Neurology Department, Faculty of Medicine, Kermanshah University of Medical Sciences, Kermanshah, Iran.; 2 Infectious Diseases Research Center, Kermanshah University of Medical Sciences, Kermanshah, Iran.; 3 Clinical Research Development Center, Imam Reza Hospital, Kermanshah University of Medical Sciences, Kermanshah, Iran.

**Keywords:** Tissue plasminogen activator, stroke, brain ischemia, stroke rehabilitation

## Abstract

**Introduction:**

Reperfusion and neuroprotection are 2 main treatment strategies exist for management of patients with ischemic stroke. This study aimed to assess the 3-month outcome of patients who underwent thrombolytic therapy following ischemic stroke.

**Methods:**

In the present prospective cohort study, the 3-month outcome of patients (mortality, disability) with acute ischemic stroke admitted to neurology department an educational hospital, Kermanshah, Iran, from 2016 to 2019, who had received thrombolytic therapy was assessed. National Institute of Health Stroke Scale (NIHSS) and Modified Rankin Score (MRS) were used for measuring the degree of disability (on admission, at the time of discharge and 3 months after thrombolytic therapy).

**Results:**

217 patients with the mean age of 66.40 ± 13.37 (27 – 97) years were studied (55.3% male). There was no significant correlation between decrease in NIHSS score and age (p = 0.44), sex (p = 0.082), time interval between initiation of symptoms (p = 0.104), and blood pressure on admission (p = 0.156). However, patients with blood sugar lower than 144 had better 3-month outcome (p = 0.045). Additionally, there was no significant correlation between the rate of decrease in MRS score and age (p = 0.813), sex (p = 0.875), time interval between initiation of symptoms (p = 0.495), and blood pressure on admission (p = 0.264). However, patients with blood sugar lower than 144 had better 3-month outcome (p = 0.022). 47 (21.7%) patient died and 170 (78.3%) were discharged. Mean age of the patients who died (73.70 ± 11.85 versus 64.39 ± 13.09 years; p < 0.0001) and their NIHSS score on admission (13.22 ± 6.01 versus 11.28 ± 5.70; p = 0.045) were significantly higher. In other words, the odds of mortality was 3.19 times in patients over 60 years of age (95% confidence interval (CI): 1.18 – 8.62) and 1.83 times in patients with NIHSS score over 12 (95% CI: 0.92 – 3.61).

**Conclusion:**

There was no significant correlation between 3-month disabilities of stroke patients underwent thrombolytic therapy and age, sex, time from initiation of symptoms, or vital signs on admission. Patients with a blood sugar lower than 144 had better 3-month outcome.

## Introduction

Stroke is the most common cause of disability and the third cause of mortality following cardiac diseases and cancer around the world. The most common type of stroke is the ischemic type. Each year about 700 thousand stroke patients are visited in the United States, 600 thousand of which are ischemic. Mortality rate of stroke is about 12% and each year 7.8 million people die of stroke all around the world and the chance of an individual having a stroke over his/her lifetime is 18%.

Previous investigations have shown that 70 – 80% of deaths due to brain stroke happen in ischemic ones (1, 2). Previously, the incidence of brain stroke among the youth was 3%; of course, this group included individuals less than 45 years of age. However, currently it can be said that this rate has increased to 7% or 8% in the same age group.

From a clinical point of view, brain stroke is associated with many kinds of problems including alterations in level of consciousness, physical, motor, cognitive, and perceptual disorders, reduced linguistic performance, pain, and psychological disorders such as depression. This has decreased improvement and rehabilitation among these patients and might even lead the patient to suicide (3).

Physiologically, brain stroke is a syndrome confirmed by acute initiation of neurological disorders persisting for at least 24 hours and is a reflection of the central nervous system being locally affected and is a result of deficiency in the brain’s blood circulation (4, 5). The aim of most existing treatments is restoring blood circulation in the ischemic penumbra area, which is the region that cells are not functional due to ischemia but if the blood circulation is restored, they will be revitalized (6, 7). Overall 2 treatment strategies exist for this disease, namely reperfusion and neuroprotection (4, 5). The most appropriate treatment presented for acute ischemic brain stroke is using arterial and venous tissue plasminogen activator (TPA) (8, 9). Ideally, more than 40% of all brain stroke patients should receive recombinant TPA (rTPA) (10-12). The present study aimed to assess the 3-month outcome of patients who underwent thrombolytic therapy following ischemic brain stroke.

## Methods

***Study design and setting***

In the present prospective cohort study, the 3-month outcome of patients with acute ischemic stroke admitted to neurology department of Imam Reza Hospital, Kermanshah, Iran, from 2016 to 2019, who had received thrombolytic therapy was assessed. The study was approved by the ethics committee of Kermanshah University of Medical Sciences (ethics code: IR.KUMS.REC.1397.642) and the researchers adhered to the principles indicated in the Declaration of Helsinki throughout the study. Before the injection of drug and inclusion in the study, written consent was obtained from the patient or their relatives.

***Participants***

All patients over 18 years of age presenting to the mentioned hospital with acute ischemic stroke, for which thrombolytic drugs were prescribed, were included. Those with hemorrhagic brain stroke, individuals for whom receiving thrombolytic drugs was contraindicated, patients whose time of stroke was unknown or had presented outside the window period of receiving thrombolytic drugs as well as those who did not give consent for participation in the study or were not available for 3-month follow-up studies were excluded from the study.

***Data gathering***

A checklist consisting of demographic data (age, sex, weight), the route of transportation to hospital, interval between initiation of symptoms to hospital presentation, underlying illnesses, blood pressure and blood sugar levels on admission, drug history, history of heart attack, National Institute of Health Stroke Scale (NIHSS) and Modified Rankin Score (MRS) (on admission, at the time of discharge and 3 months after thrombolytic therapy), duration of hospitalization, and in-hospital mortality was filled out for all of the patients by a senior neurology resident. All the patients were re-evaluated using NIHSS and MRS scores 3 months after thrombolytic therapy. To invite the patients for examination 3 months later, they were contacted via phone and were invited to the clinic.

***Outcomes***

The evaluated outcomes in the present study consisted of 3-month mortality and disability of the patients and NIHSS and MRS indices were used to perform the evaluations.

***TPA injection method***

The precise time of initiation of the first symptom (including dysarthria, hemiparesis, and …) was asked from the patients or their relatives and a thorough history was taken regarding heart attack, stroke, and medications used as well as risk factors including hypertension, diabetes, and smoking. Blood examinations including glucometry and brain computed tomography (CT) scan were also performed. In case of hypertension, respiratory disorder and hemodynamic instability, the patient was stabilized first. If hemorrhage was not detected in brain CT scan and no contraindication was present, thrombolytic injection was done. The dose of the drug was 0.9 mg/kg body weight, 10% of which was administered as a bolus dose and the rest was infused over 1 hour. In the present study, the medication used was alteplase manufactured by Boehringer Ingelheim Company. All injections were done under complete cardiorespiratory monitoring and direct supervision of the in-charge neurologist. In addition, all the CT scans were also interpreted by the in-charge neurologist.

***NIHSS***

It is used to evaluate the effect of acute brain stroke on level of consciousness, language, attention, field of vision, eye movement, muscular power, speech, sensory function, and ataxia. This scale has 15 items that should be graded based on neurological examination. Based on the patient’s answers and ability to move in each item, the examiner gives the patient a final score ranging from 0 to 42, in which zero indicates being normal (13).

***MRS***

A useful tool for measuring the degree of disability and dependence in the daily activities of those who suffer from brain damage. Validity and reliability of this scale has been proved. The score range of this tool is 0 to 6. Score of 0 indicates absence of any sign of neurological damage. Score of 1 shows that the patient does not have a considerable disability and is able to do his/her regular activities and duties. Score of 2 means that the patient has mild disability and cannot perform tasks like before, but can take care of him/herself without help. Score of 3 indicates that the patient has moderate disability and needs help, but can walk without any help. Score of 4 shows that the patient has relatively severe disability and cannot walk without help, and cannot survive without help. Score of 5 means that patient has relatively severe disability and is bedridden, has incontinence and requires constant care and nursing. Score of 6 indicates death of the patient (14-16).

***Statistical analysis***

Extracted data were entered to SPSS software version 21 and underwent statistical analysis. Quantitative variables were reported as mean and standard deviation, and qualitative ones were reported as frequency and percentage. T test, chi-square and Fisher exact tests were used for comparisons. P < 0.05 considered as level of significance. 

## Results

***Baseline characteristics of the studied patients***

217 patients with the mean age of 66.40 ± 13.37 (27 – 97) years and average weight of 70.74 ± 11.99 (50 – 120) kg were studied (55.3% male). Table 1 depicts the baseline characteristics of the studied patients. Mean time interval between initiation of the symptoms and presentation to the hospital was 112.75 ± 58.24 (15 – 240) minutes. In addition, mean blood sugar of the patients was 138.82 ± 65.59 mg/dl (using glucometer) on admission. Mean time interval between admission and receiving thrombolytic therapy was estimated to be 39.02 ± 19.4 (8 – 120) minutes.

***Outcomes***

On average, patients were hospitalized for 13.25 ± 13.48 (1-74) days. Mean NIHSS (p < 0.001) and MRS (p < 0.001) scores had significantly improved at the time of discharge and 3 months after the treatment (table 2).

**Table 1:** Baseline characteristics of the studied patient

Variable	**Value **
**Sex **	
Male	120 (55.3)
Female	97 (44.7)
**Age (year)**	
< 60	53 (24.4)
≥ 60	164 (75.6)
**Route of transportation to hospital**	
Personal vehicle	162 (74.7)
Ambulance	115 (25.3)
**Symptom initiation to reaching hospital (minute) **	
≤ 180	183 (84.3)
> 180	34 (15.7)
**Blood pressure on admission (mmHg)**	
Systolic	146.10 ± 24.48
Diastolic	89.17 ± 18.1
**History of disease**	
Hypertension	144 (73.0)
Diabetes	25 (11.5)
Brain stroke	23 (10.6)
Hyperlipidemia	6 (2.8)
Atrial fibrillation	19 (8.8)
**Smoking **	
No	194 (89.4)
Yes	23 (10.6)
**Coagulant use **	
No	194 (89.4)
Yes	23 (10.6)
**Disability ** **on admission**	
NIHSS	11.69 ± 5.81
MRS	2.72 ± 1.9

Data are presented as frequency (%) or mean ± standard deviation. NIHSS: National Institute of Health Stroke Scale; MRS: Modified Rankin Score.

**Table 2:** Mean scores of the patients in National Institute of Health Stroke Scale (NIHSS) and Modified Rankin Score (MRS) indices on admission, at the time of discharge and 3 month after treatment

**Index **	**Time of measurement**			**P**
	**On admission**	**Discharge**	**3 months later**	
NIHSS	11.69 ± 5.81	6.35 ± 4.61	5.47 ± 4.48	< 0.001
MRS	2.72 ± 1.9	2.47 ± 1.32	2.18 ± 1.44	< 0.001

There was no significant correlation between decrease in NIHSS score and age (p = 0.44), sex (p = 0.082), time interval between initiation of symptoms and presenting to the hospital (p = 0.104), and blood pressure on admission (p = 0.156). However, there was a significant correlation between decrease in NIHSS score in patients and blood sugar under 144; patients with blood sugar lower than 144 had better 3-month outcome (p = 0.045).

Additionally, there was no significant correlation between the rate of decrease in MRS score and age (p = 0.813), sex (p = 0.875), time interval between initiation of symptoms and presenting to the hospital (p = 0.495), and blood pressure on admission (p = 0.264). However, there was a significant correlation between decrease in MRS score in patients and blood sugar under 144; patients with blood sugar lower than 144 had better 3-month outcome (p = 0.022).

47 (21.7%) patient died and 170 (78.3%) were discharged. Mean age of the patients who died (73.70 ± 11.85 versus 64.39 ± 13.09 years; p < 0.0001) and their NIHSS score on admission (13.22 ± 6.01 versus 11.28 ± 5.70; p = 0.045) were significantly higher. In other words, the odds of mortality was 3.19 times in patients over 60 years of age (95% confidence interval (CI): 1.18 – 8.62) and 1.83 times in patients with NIHSS score over 12 (95% CI: 0.92 – 3.61).

## Discussion

Based on the findings of the present study, the rate of in-hospital mortality of patients with brain stroke undergoing thrombolytic therapy was estimated to be about 22% and age over 60 years and NIHSS over 12 on admission were the significant risk factors of death. There was no significant correlation between 3-month outcome of the patients regarding disability based on NIHSS and MRS indices and demographic data (age and sex), time from initiation of symptoms, or vital signs on admission. Patients with a blood sugar lower than 144 had better 3-month prognosis based on the mentioned scales. 

In 2016, Baratloo et al. showed that 30% of the brain stroke patients who met the criteria of thrombolytic therapy had received rTPA in Iran. High cost of rTPA and lack of proper infrastructure were mentioned as the major obstacles for thrombolytic therapy in that study (17).

 The results of Dong et al. (2015), which were in line with the present study, indicated that mean NIHSS and MRS scores and hospitalization duration significantly decreased in patients with ischemic stroke who received venous TPA (18). This finding has also been confirmed in the study by Tosta et al. (19).

Mehta et al. (2017) also presented results in line with the present study, indicating that in 3-month follow-up, an acceptable improvement is found in 67% of patients receiving rTPA. 32% of the patients deteriorated and severity of symptoms on admission, blood sugar on admission, age and type of stroke affected the outcome of patients (20).

In the study by Albers et al. (2000) the rate of 30-day mortality was 13% among patients receiving rTPA, 35% of the patients had considerably improved and had MRS scores less than 1 and 43% of them were independent in their daily activities and had MRS scores less than 2. Patients with initial NIHSS over 10 showed less improvement and there was a significant correlation between increase in the patient’s blood pressure on admission and decrease in probability of improvement (21). However, in the present study no significant correlation was found between patients’ blood pressure on admission and the rate of improvement in 3-month outcome.

The reason for the differences in factors found to affect the outcome of stroke patients in various studies could be differences in duration of follow-up, baseline characteristics of the studied patients, race, medications used, and care provided after thrombolytic therapy. On the other hand, in these studies it is often not known if the patients have undergone rehabilitation and physiotherapy or not and therefore, to reach better conclusions, further controlled studies might be needed.

Considering the results of the present study, it seems that paying more attention to patients with higher risk and screening them, providing more care and more regularly monitoring them may be effective in decreasing their mortality. However, it should be noted that based on the findings of the present study and other existing studies, most introduced risk factors affecting the outcome of these patients such as age, underlying illness, blood pressure, and etc. cannot be controlled by the physician. Therefore, in this regard, the best level of prevention might be the primary level, which is controlling non-contagious diseases such as hypertension and diabetes.

***Limitations***

Small sample size, being single centered, short duration of the follow-up period, and not checking other factors affecting patients’ outcome such as presence or absence of rehabilitation are among the limitations of the present study.

## Conclusion

Based on the findings of the present study, the rate of in-hospital mortality of patients with brain stroke undergoing thrombolytic therapy was estimated to be about 22% and age over 60 years and NIHSS over 12 on admission were the only significant risk factors of death. There was no significant correlation between 3-month outcome of the patients regarding disability based on NIHSS and MRS indices and demographic data (age and sex), time from initiation of symptoms, or vital signs on admission. Patients with a blood sugar lower than 144 had better 3-month prognosis based on the mentioned scales. 
